# Chelation Therapy with Oral Solution of Deferiprone in Transfusional Iron-Overloaded Children with Hemoglobinopathies

**DOI:** 10.1155/2013/121762

**Published:** 2013-11-04

**Authors:** Alexandros Makis, Nikolaos Chaliasos, Sapfo Alfantaki, Paraskevi Karagouni, Antigone Siamopoulou

**Affiliations:** ^1^Department of Pediatrics, University Hospital of Ioannina, Stavros Niarchos Avenue, 45500 Ioannina, Greece; ^2^Child Health Department, University of Ioannina Medical School, P.O. Box 1187, 45110 Ioannina, Greece

## Abstract

Iron overload in hemoglobinopathies is secondary to blood transfusions, chronic hemolysis, and increased iron absorption and leads to tissue injury requiring the early use of chelating agents. The available agents are parenteral deferoxamine and oral deferiprone and deferasirox. There are limited data on the safety and efficacy of deferiprone at a very young age. The aim of our study was the presentation of data regarding the use of oral solution of deferiprone in 9 children (mean age 6.5, range 2–10) with transfusion dependent hemoglobinopathies (6 beta thalassemia major, 1 thalassemia intermedia, and 2 sickle cell beta thalassemia). The mean duration of treatment was 21.5 months (range 15–31). All children received the oral solution without any problems of compliance. Adverse reactions were temporary abdominal discomfort and diarrhea (1 child), mild neutropenia (1 child) that resolved with no need of discontinuation of treatment, and transient arthralgia (1 child) that resolved spontaneously. The mean ferritin levels were significantly reduced at the end of 12 months (initial 2440 versus final 1420 **μ**g/L, *P* < 0.001). This small study shows that oral solution of deferiprone was well tolerated by young children and its use was not associated with major safety concerns. Furthermore, it was effective in decreasing serum ferritin.

## 1. Introduction

Children with transfusion dependent hemoglobinopathies rapidly develop potentially damaging levels of toxic iron overload in many vital organs. Iron removal with chelating agents is required early in life and in some cases after the age of two [1]. Therefore, the safety, efficacy, and compliance regarding the use of iron chelators are of high significance because of the long-term nature of the treatment. Currently available chelators are deferoxamine, which is administered intravenously or subcutaneously, and the deferasirox and deferiprone that are given orally. The oral administration of iron chelation is most welcome by children with hemoglobinopathies who have problems with the discomfort of deferoxamine injections. Furthermore, early and aggressive chronic administration with deferoxamine can affect many organs and especially skeletal maturation and result in growth retardation [2, 3]. But even more important is deferiprone's superior ability to protect the heart, which has been shown to improve survival in many studies [4, 5]. Limited clinical data on the safety and efficacy of deferiprone on very young age allow us to add our experience [6–8].

The aim of our study was the presentation of data regarding the clinical benefit and side effects of liquid oral solution of deferiprone in young children with hemoglobinopathies less than 10 years old.

## 2. Patients and Methods

The Thalassemia Unit of the Department of Pediatrics at the University Hospital of Ioannina, Ioannina, Greece, is the reference center for children with hemoglobinopathies in northwest Greece and patients regularly attend for investigation, clinical follow-up, and treatment; their information is prospectively recorded. We collected information during the treatment with liquid oral solution of deferiprone (Ferriprox 100 mg/mL) from nine children with transfusion dependent hemoglobinopathies. Six children had beta thalassemia major, 1 thalassemia intermedia, and 2 sickle cell beta thalassemia. The principles outlined in the Declaration of Helsinki had been followed. The diagnosis was based on the clinical, hematological, biosynthetic, and genetic studies. The characteristics of the study group are shown in Table 1. The mean number of red blood cell transfusions during the previous year was 10.6 (range 8–15). All the children had chronic iron overload requiring chelation therapy, as defined by serum ferritin concentration (mean ± standard error (SE): 2440 ± 1275 *μ*g/L). The mean age at the beginning of the treatment was 6.5 (range 2–10), five were boys, and four were girls. All the children were naïve to iron chelation therapy before this study, except two patients who were receiving deferoxamine (*N* = 2; mean dose = 35 mg/kg/d; mean duration of use = 1.5 years). One child was splenectomized. All the children had negative anti-hepatitis C antibody status at baseline.

Therapy, with oral solution of deferiprone, was initiated at a daily dose of 50 mg/kg, divided into 3 doses, for the first 2 weeks. The dose was increased to 75 mg/kg, for another 2 weeks. If the patient's serum ferritin concentration at baseline was greater than 2500 *μ*g/L, the dose was further increased to a total daily dose of 100 mg/kg after 4 weeks of deferiprone therapy. After initiation of the treatment, full blood count was assessed weekly, serum ferritin was assessed monthly, and liver and renal functions were assessed bimonthly. The mean duration of the administration of liquid oral solution of deferiprone was 21.5 months (range 15–31 months). To evaluate the efficacy and safety of deferiprone treatment, biochemical parameters such as serum ferritin and liver enzymes were analyzed using the Student *t*-test. All parameters are presented as mean ± standard error (SE). *P* values less than 0.05 were considered statistically significant.

## 3. Results and Discussion

**Figure 1:**
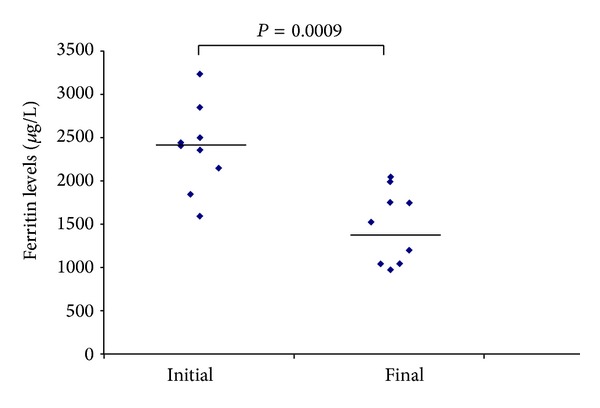
Serum ferritin concentrations during treatment with oral solution of deferiprone (Ferriprox 100 mg/mL) in nine children with transfusion dependent hemoglobinopathies.

The hematological and biochemical markers during treatment are shown in Table 2. All the children received the oral deferiprone without any problems of compliance. The adverse reactions that were noticed during treatment were the following: temporary abdominal discomfort and diarrhea at initiation of therapy that resolved despite continued deferiprone (one child, age 8 years), mild neutropenia (1.2 × 10^9^/L) that resolved within 8 days despite continued deferiprone therapy (one child, age 4 years), and transient arthralgia that resolved spontaneously (one child, age 10). Deferiprone oral solution was effective in reducing serum ferritin (mean ± SE) (initial 2440 ± 1275 *μ*g/L versus final 1420 ± 730 *μ*g/L, *P* < 0.001) (Figure 1). Five children of the study were <6 years old. The baseline serum ferritin of these children was significantly lower than the children >6 years (2250 *μ*g/L ±880 versus 2950 *μ*g/L ±1550, *P* < 0.005). The differences in changes in serum ferritin as well as in other parameters did not reach statistical significance between the two age groups (Table 2).

Deferiprone, previously only in tablet form, is not suitable for young children under the age of six years. Recently the solution form of deferiprone has been introduced. However, the clinical data regarding its use in small children are quite limited. A very recent clinical study of deferiprone on children less than 10 years was conducted in Egypt, Malaysia, and Indonesia [8]. El Alfy and colleagues reviewed the use of liquid oral solution of deferiprone in 100 children (mean age 5.1 years) with thalassemia or sickle cell disease. At the end of the 6-month treatment period, there was a significant drop in mean serum ferritin levels. Therapy was not associated with unexpected adverse reactions, as it was noticed in our study. Similar results were reported in another trial of pediatric patients who received tablet formulation of deferiprone (median age 10.6 years) [7]. Mean serum ferritin levels decreased significantly following a median treatment duration of 11.4 months. The investigators of another study tried to assess the safety of deferiprone in very young children (<6 years). Nausea and vomiting were noticed in 27%, joint symptoms in 9%, and neutropenia in 4.5% of the patients. None of the patients had agranulocytosis. Interestingly, thrombocytopenia was observed in 45% of the patients. Transient interruption of treatment led to reversal of symptoms in the majority of patients [6].

In our study, we examined the clinical benefit and safety of liquid oral solution of deferiprone in young children with transfusion dependent hemoglobinopathies. Although the number of the patients was small, we noticed that deferiprone oral solution was well tolerated and its use was not associated with major safety concerns, while it was effective in decreasing serum ferritin.

In conclusion, assuming the same biological efficacy as the tablet form, deferiprone solution seems to benefit the young children, and they may not require injectable iron chelation. Further studies with large number of patients and long-term follow-up are needed to evaluate the safety and efficacy profile of deferiprone oral solution in small children.

## Figures and Tables

**Table 1:** Demographic and clinical characteristics of nine children with transfusion dependent hemoglobinopathies that received oral solution of deferiprone (Ferriprox 100 mg/mL).

Characteristics	*N*
Age (years)	2–10, mean 6.5
Male : Female	5 : 4
*β*-Thalassemia major	6
Thalassemia intermedia	2
Sickle/*β* thalassemia	1
Red blood cell transfusions during the last year	Range 8–15, mean 10.6
Chelation therapy with deferoxamine before the initiation of the study	*N* = 2; mean dose = 35 mg/kg/d; mean duration of use = 1.5 years
Splenectomy	1
Oral liquid deferiprone dosage	50–100 mg/Kg/d, mean 75.2
Duration of oral liquid deferiprone (months)	Range 15–31, mean 21.5

**Table 2:** Hematological and biochemical markers during treatment with oral solution of deferiprone (Ferriprox 100 mg/mL) in nine children (4 under 6 years, 5 above 6 years) with transfusion dependent hemoglobinopathies.

Parameters	Total		<6 years		>6 years	
	Initial	Final	Initial	Final	Initial	Final
White blood cells (×10^9^/L)	6.5 ± 0.6	6.7 ± 1.2	5.9 ± 1.4	6.3 ± 2.1	6.8 ± 1.3	6.1 ± 0.8
Hemoglobin (g/L)	95 ± 8	94 ± 6	89 ± 9	93 ± 7	97 ± 1.1	93 ± 7
Platelets (×10^9^/L)	178.2 ± 56.9	165.4 ± 58.4	182.4 ± 91.8	175.5 ± 56.2	174.8 ± 66.3	180.7 ± 95.7
AST (U/L)	43.5 ± 15.4	39.2 ± 14.6	42.3 ± 17.5	39.7 ± 20.5	38.9 ± 13.8	41.8 ± 13.7
ALT (U/L)	53.1 ± 16.5	51.4 ± 13.7	50.8 ± 12.9	49.8 ± 19.1	54.2 ± 17.2	51.8 ± 15.4
Creatinine (*μ*mol/L)	65 ± 12	72 ± 15	68 ± 12	70 ± 11	68 ± 19	72 ± 16
Ferritin (*μ*g/L)	2440 ± 1275	1420 ± 730*	2250 ± 880	1575 ± 810*	2950 ± 1550	1390 ± 985*

Values presented as mean ± SE; *P < 0.05.
